# Risk factors associated with mortality in patients hospitalized for coronavirus disease 2019 in Rio de Janeiro, Brazil

**DOI:** 10.1590/0037-8682-0878-2020

**Published:** 2021-03-22

**Authors:** Julio César Delgado Correal, Victor Edgar Fiestas Solórzano, Paula Hesselberg Damasco, Maria de Lourdes Martins, Adriana Guerreiro Soares de Oliveira, Carla Salles Campos, Marcos Fernando Fornasari, Elzinandes Leal de Azeredo, Paulo Vieira Damasco

**Affiliations:** 1 Hospital Rio Botafogo, Rio de Janeiro, RJ, Brasil.; 2 Fundação Oswaldo Cruz, Instituto Oswaldo Cruz, Rio de Janeiro, RJ, Brasil.; 3 Universidade Federal Fluminense, Niterói, RJ, Brasil.; 4 Universidade do Estado do Rio de Janeiro, Rio de Janeiro, RJ, Brasil.; 5 Universidade Federal do Estado do Rio de Janeiro, Rio de Janeiro, RJ, Brasil.

**Keywords:** COVID-19, Kaplan-Meier analysis, Mortality, Brazil

## Abstract

**INTRODUCTION:**

Understanding the mortality-associated risk factors of coronavirus disease 2019 will impact clinical decisions.

**METHODS:**

This retrospective longitudinal study included patients hospitalized for coronavirus disease in Rio de Janeiro, Brazil. The Kaplan-Meier method and multivariate Cox regression analysis were used.

**RESULTS:**

Sequential Organ Failure Assessment score of ≥2 (hazard ratio 4.614; 95% confidence interval =2.210-9.634; p<0.001) and neutrophil/lymphocyte ratio of >5 (hazard ratio=2.616; 95% confidence interval=1.303-5.252; p=0.007) were independently associated with mortality.

**CONCLUSIONS:**

Sequential Organ Failure Assessment score and neutrophil/lymphocyte ratio on admission can identify coronavirus disease patients at increased risk of death and guide subsequent clinical decisions.

Severe acute respiratory syndrome coronavirus 2 (SARS-CoV-2) is a viral pathogen that rapidly caused a devastating pandemic of coronavirus disease 2019 (COVID-19). As of December 12, 2020, SARS-CoV-2 has caused 71,704,885 infections and 1,604,978 deaths worldwide^1^. On February 26, 2020, the Ministry of Health confirmed the first case of COVID-19 in Brazil. Almost a year after the pandemic, Brazil has the third-highest number of confirmed cases and the second-most deaths in the world^1^ ^-^ ^2^.

Even though the majority of patients who develop COVID- 19 have mild symptoms^3^, about 20% of hospitalized patients are admitted to an intensive care unit (ICU), 15% require mechanical ventilation (MV), and up to 20% of hospitalized patients die^4^ ^,^ ^5^. Studies on risk factors for intubation and mortality in patients hospitalized for COVID-19 have largely focused on patients from China, Europe, and the United States of America^6^. 

In contrast, in Latin America, the hospitalized patients’ characteristics, comorbidities, presenting symptoms, laboratory parameters, and clinical outcomes have not been thoroughly investigated. Considering that it has not yet been clarified whether phenotypic and genotypic characteristics of the population cause differences in the response to SARS-CoV-2 infection^7^, understanding the factors associated with mortality in patients with COVID-19 could impact clinical decisions and guide public health policies in Latin American countries.

We conducted a retrospective longitudinal study that included adult patients admitted to Hospital Casa Rio-Botafogo with suspected or confirmed COVID-19, according to the clinical, radiological, and laboratory criteria of the Ministry of Health-Brazil^8^, and those who died or were discharged between March 4, 2020 and June 21, 2020. This reference hospital was a private one that was located in the city of Rio de Janeiro, Brazil.

Using a form prepared for the study that was validated with the hospital file, general information was obtained from the reports of the Emergency Unit, and variables recorded during the physical examinations and laboratory tests performed on admission were also collected. In addition, information on hospitalization was obtained, including length of stay, admission to the ICU, treatment, use of MV, complications, and outcome (discharge/death).

The final number of patients included in the study was 98 after excluding five patients due to incomplete information, and the missing data were not imputed. For the descriptive analysis, continuous variables are presented as means with standard deviations (SDs) or as medians with interquartile ranges (IQRs), as appropriate. Univariate analyses to identify variables associated with the outcome (discharge/death) were performed using the Chi square test, Fisher exact test, student’s t-test, or Mann-Whitney U test, as appropriate.

The Kaplan-Meier method was used for the survival analysis. The time calculated from the date of hospital admission to the date of death or last day of hospitalization was considered as a dependent variable. The log-rank test was used to compare the survival curves, and the Cox proportional-hazards model was used to identify the baseline variables that were independently associated with death at hospital admission after adjustments for age, sex, and comorbidities. Schoenfeld residue analysis was performed to verify the proportionality of the risks, and Cox-Snell residue analysis was used to adjust the models. The analyses were conducted using STATA version 15.0 (StataCorp, College Station, TX, USA) and statistical significance was set at p<0.05.

The study was approved by the Ethics Research Committee of Hospital Casa Rio-Botafogo in July 2020 and the CONEP Brazil Platform under CAAE number 47885515.8.0000.5279. All procedures were performed in accordance with the principles of Declaration of Helsinki, 1964, as revised in 1975, 1983, 1989, 1996, and 2000.

More than half of the patients (51%) were men aged >70 years, and the majority (91%) had at least one comorbidity, with the most frequent being hypertension (72%), diabetes mellitus (30%), obesity (21%), and stroke (19%). A considerable proportion lived in nursing homes (20%) and had been previously hospitalized in the last 3 months (21%) (Table 1). 

On admission, the most frequent symptoms were cough (81%), dyspnea (77%), and fatigue (51%) (Table 2). The highest number of hospitalizations (79%) and deaths (75%) occurred between epidemiological weeks 16 and 20, which coincided with that observed in the metropolitan region of Rio de Janeiro^2^ (Supplemental **Figure 1**).

**TABLE 1:** Demographic and baseline characteristics of the patients.

	Total		Survived		Died		p
	n	%	n	%	n	%	
Age* (years)	71 (56, 86)		68 (53, 80)		75 (63, 88)		**0.039**
Sex							
Male	50	51.0	25	46.3	25	56.8	0.300
Female	48	49.0	29	53.7	19	43.2	
Comorbidity	89	90.8	47	87.0	42	95.5	0.180
Hypertension	71	72.4	38	70.4	33	75.0	0.610
Diabetes mellitus	29	29.6	14	25.9	15	34.1	0.378
Obesity	21	21.4	8	14.8	13	29.5	0.077
Stroke	19	19.4	6	11.1	13	29.5	**0.022**
Hypothyroidism	15	15.3	7	13.0	8	18.2	0.475
COPD	10	10.2	7	13.0	3	6.8	0.504
Atrial fibrillation	10	10.2	5	9.3	5	11.4	0.750
Congestive heart failure	10	10.2	4	7.4	6	13.6	0.337
Chronic kidney disease	10	10.2	5	9.3	5	11.4	0.750
Cancer	10	10.2	6	11.1	4	9.1	1.000
Immunosuppression	7	7.1	3	5.6	4	9.1	0.697
Coronary artery disease	7	7.1	2	3.7	5	11.4	0.238
Dyslipidemia	3	3.1	0	0.0	3	6.8	0.087
Asthma	3	3.1	2	3.7	1	2.3	1.000
Dementia	2	2.0	1	1.9	1	2.3	1.000
Myocardial infarction	1	1.0	0	0.0	1	2.3	0.449
HIV infection	1	1.0	0	0.0	1	2.3	0.449
Charlson Comorbidity Index**	3.6 ± 2.1		3.2 ± 2.3		4.2 ± 1.8		**0.018**
Prior hospitalization in the last 3 months	21	21.4	12	22.2	9	20.5	0.832
Nursing home resident	20	20.4	8	14.8	12	27.3	0.128
Prior pneumonia episode	4	4.1	1	1.9	3	6.8	0.323

(*) median (IQR); (**) mean ± standard deviation (SD); **COPD:** chronic obstructive pulmonary disease; **HIV:** human immunodeficiency.

**TABLE 2:** Clinical characteristics and laboratory parameters of the patients on admission to the hospital.

	Total		Survived		Died		p
	n	%	n	%	n	%	
**Clinical characteristics**							
Cough	79	80.6	46	85.2	33	75.0	0.205
Dyspnea	75	76.5	42	77.8	33	75.0	0.747
Fatigue	50	51.0	25	46.3	25	56.8	0.300
Myalgia	39	39.8	20	37.0	19	43.2	0.536
Coryza	13	13.3	7	13.0	6	13.6	0.922
Pharyngitis	11	11.2	7	13.0	4	9.1	0.750
Diarrhea	7	7.1	5	9.3	2	4.5	0.454
Temperature* (°C)	36.7	(36.5, 37.4)	36.7	(36.5, 37.2)	37	(36.6, 37.7)	0.157
Heart rate*	82	(72, 93)	81	(72, 92)	84	(71, 96)	0.471
Respiratory rate*	22	(18, 24)	22	(18, 24)	22	(19, 24)	0.320
APACHE II score*	6	(6, 8)	6	(6, 8)	8	(6, 12)	**0.001**
SOFA score*	1	(1, 2)	1	(1, 2)	2	(1, 3)	**<0.001**
Glasgow coma scale*	15	(11, 15)	15	(14, 15)	12	(9, 15)	**<0.001**
Mean arterial pressure*	78	(75, 90)	79	(75, 92)	75	(69, 90)	**0.028**
<70 mm Hg	13	13.3	2	3.7	11	25.0	**0.002**
Oxygen saturation*	96	(92, 98)	96	(94, 98)	93	(90, 97)	**0.002**
<95%	41	41.8	16	29.6	25	56.8	**0.007**
**Laboratory parameters**							
Hemoglobin** (mg/dL)	11.6 ± 2.0		11.8 ± 2.1		11.3 ± 2.0		0.236
Hematocrit** (%)	34.1 ± 6.2		34.5 ± 6.5		33.7 ± 5.8		0.532
Leucocytes* (x mm^3^)	7,900	(5,375, 11,900)	6,550	(5,100, 8,900)	10,750	(7,100, 15,100)	**<0.001**
Neutrophils* (x mm^3^)	6,300	(4,158, 9,520)	4898	(3,680, 7,144)	8,970	(5,822, 11,830)	**<0.001**
Lymphocytes* (x mm^3^)	1,188	(846, 1,775)	1,287	(945, 1,768)	987	(779, 1,860)	0.271
Monocytes* (x mm^3^)	420	(270, 564)	389	(264, 505)	445	(348, 650)	0.112
Platelets* (x 10^3^/mm^3^)	224	(157, 301)	220	(157, 279)	238	(157, 354)	0.292
NLR	5.3	(3.3, 8.2)	3.8	(2.6, 6.6)	6.6	(4.9, 11)	**<0.001**
MLR	0.3	(0.3, 0.5)	0.3	(0.2, 0.4)	0.4	(0.3, 0.6)	**0.027**
PLR	179.1	(121.7, 253)	169.8	(129, 230.3)	208.2	(107.3, 287.6)	0.130
Glucose* (mg/dL)	125	(115, 140)	120	(110, 135)	125	(115, 145)	0.056
BUN* (mg/dL)	48	(31, 94)	45	(30, 62)	76	(35, 145)	**0.025**
Creatinine* (mg/dL)	1.1	(0.7-1.9)	1.1	(0.7-1.5)	1.3	(0.7-2.8)	0.079
LDH* (U/L)	838	(548, 1,050)	620	(455, 1,007)	960	(604, 1,161)	**0.017**
Albumin** (g/dL)	2.8 ± 0.5		3.0 ± 0.5		2.6 ± 0.5		**0.001**
Total bilirubin* (mg/dL)	0.5	(0.3-0.7)	0.4	(0.3-0.6)	0.6	(0.4-1.0)	**0.001**
AST* (U/L)	61	(35-102)	55	(28-75)	70	(47-111)	**0.029**
ALT* (U/L)	47	(25-79)	35	(21-86)	54	(33-77)	0.301
C-reactive protein* (mg/L)	9	(5.4, 18.5)	7.8	(3.7, 11.5)	16.8	(6.9, 26.6)	**0.001**

(*) median (IQR); (**) mean ± standard deviation (SD); **APACHE:** Acute Physiology and Chronic Health Evaluation; **SOFA:** Sequential Organ Failure Assessment; **NLR:** neutrophil/lymphocyte ratio; **MLR:** monocyte/lymphocyte ratio; **PLR:** platelet/lymphocyte ratio; **BUN:** blood urea nitrogen; **LDH:** lactate dehydrogenase; **AST:** aspartate aminotransferase; **ALT:** alanine aminotransferase.

A higher proportion of statistically significant deaths was found in older patients (p = 0.039), those with a higher Charlson comorbidity index (p = 0.018), and those with a history of stroke (p = 0.022). However, we also found a higher proportion of deaths in patients who lived in nursing homes (60%, 12/20).

In addition, the following parameters were found at hospital admission more frequently in patients who died: a mean arterial pressure of <70 mm Hg (p = 0.002); peripheral oxygen saturation of <95% (p = 0.007); higher leukocyte value (p<0.001), neutrophil count (p<0.001), neutrophil/lymphocyte ratio (NLR) (p<0.001), monocyte/lymphocyte ratio (MLR) (p = 0.027), blood urea nitrogen (BUN) level (p = 0.025), lactate dehydrogenase level (LDH) (p = 0.017), total bilirubin level (p = 0.001), aspartate aminotransferase (AST) level (p = 0.029), C-reactive protein (CRP) level (p = 0.001); lower serum albumin level (p = 0.001); a higher Acute Physiology and Chronic Health Disease (APACHE) II score (p = 0.001) and Sequential Organ Failure Assessment (SOFA) score (p<0.001); and a lower value on the Glasgow scale (p<0.001) (Table 2).

SARS-CoV-2 infection was confirmed in the laboratory by real-time reverse transcription polymerase chain reaction (RT-PCR) in 58 patients (59%). Co-infection with influenza A (H1N1) virus was confirmed in two 75-year-old patients who died. In addition, a 53-year-old woman with human immunodeficiency virus (HIV) infection, but no other comorbidities, died.

Most patients (86%) were admitted to the ICU. MV, hemodialysis, and blood transfusion were more associated with the group of patients who died; however, only MV was statistically significant (p<0.001). On the other hand, sepsis (98%), septic shock (98%), acute respiratory distress syndrome (91%), acute renal failure (84%), acute liver failure (52%), disseminated intravascular coagulation (36%), and encephalitis (27%) were more frequently associated with mortality (Supplemental **Table 1**).

At the time of observation, there were 44 deaths (45%) and a mortality rate of 38.2 (95% confidence interval [CI], 28.3-51.4) per 1000 patient-days. Death after 48 hours, 7 days, and 30 days of admission occurred in 3 (7%), 23 (52%), and 42 patients (96%), respectively. All those who were not admitted to the ICU and 40 patients (48%) who were admitted to the ICU survived. 

Table 3 shows the mortality rate per 1000 patient-days and estimated hazard ratio (HR) of death for the demographic, clinical, and laboratory variables at admission that were significant in the univariate analysis. We observed that age >70 years, history of stroke, Charlson comorbidity index ≥4, APACHE II score ≥4, SOFA score ≥2, Glasgow Coma scale <15, mean arterial pressure <70 mm Hg, peripheral oxygen saturation <95%, NLR >5, LDH level >840 U/L, albumin level <3 g/dL, total bilirubin level >0.5 mg/dL, AST level >50 U/L, and CRP level >9 mg/L were associated with an increased risk of mortality. In the multivariable stepwise Cox regression model, only a SOFA score of ≥2 (HR = 4.614, 95% CI = 2.210-9.634, p<0.001) and an NLR of >5 (HR = 2.616, 95% CI = 1.303-5.252, p = 0.007) were independently associated with mortality (**Supplemental** Figures 2 **and** 3).

**TABLE 3:** Mortality rates and hazard ratios associated with the baseline clinical and laboratory variables of the patients.

	Mortality rate per 1000 patient-days (95% CI)		HR (95% CI)		p
**Age**					
≤70 years	33.5	(17.4-64.3)			
70 years	45.3	(31.1-66.1)	1.462	(0.786-2.718)	0.220
**Stroke**					
No	36.7	(25.6-52.5)			
Yes	42.1	(24.4-72.5)	1.278	(0.658-2.484)	0.461
**Charlson Comorbidity Index**					
0-3	29.4	(17.4-49.6)			
≥4	44.6	(31.0-64.2)	1.426	(0.751-2.708)	0.267
**APACHE II score**					
0-6	27.1	(17.3-42.5)			
≥7	56.3	(37.8-84.1)	**1.995**	**(1.077-3.696)**	**0.023**
**SOFA score**					
0-1	22.0	(13.0-37.2)			
≥2	59.1	(41.0-85.0)	**2.602**	**(1.348-5.022)**	**0.003**
**Glasgow Coma scale**					
15	29.3	(18.2-47.1)			
<15	47.6	(32.4-69.9)	1.645	(0.884-3.063)	0.107
**Mean arterial pressure**					
≥70 mm Hg	31.6	(22.4-44.7)			
<70 mm Hg	95.7	(53.0-172.7)	**2.948**	**(1.469-5.916)**	**0.001**
**Oxygen saturation (%)**					
100%-95%	26.3	(16.8-41.3)			
<95%	59.3	(39.7-88.4)	**2.206**	**(1.192-4.083)**	**0.009**
**NLR**					
≤5	26.5	(15.4-45.6)			
>5	47.2	(33.0-67.5)	**2.064**	**(1.067-3.994)**	**0.025**
**Lactate dehydrogenase (U/L)**					
≤840	28.3	(16.4-48.7)			
>840	45.0	(31.4-64.3)	1.655	(0.855-3.205)	0.124
**Albumin (g/dL)**					
≥3	29.3	(18.9-45.5)			
<3	50.8	(34.3-77.8)	1.649	(0.904-3.007)	0.094
Total bilirubin (mg/dL)					
≤0.5	26.7	(16.6-43.0)			
>0.5	53.0	(36.1-77.8)	2.263	(1.216-4.211)	0.007
AST (U/L)					
≤50	32.7	(20.0-53.3)			
>50	42.4	(29.1-61.8)	1.284	(0.689-2.392)	0.423
C-reactive protein (mg/L)					
≤9	26.1	(15.8-43.3)			
>9	50.6	(35.0-73.3)	1.991	(1.056-3.754)	0.027

**CI:** confidence interval; **HR:** hazard ratio; **APACHE:** Acute Physiology and Chronic Health Evaluation; **SOFA:** Sequential Organ Failure Assessment; **NLR:** neutrophil/lymphocyte ratio; **AST:** aspartate aminotransferase.

Rio de Janeiro state has the fourth-highest number of COVID-19-confirmed cases in Brazil, behind only the states of São Paulo, Bahia, and Minas Gerais. However, in terms of absolute number of deaths, Rio de Janeiro ranks second, below the state of São Paulo^2^. To the best of our knowledge, this is the first study to evaluate the factors associated with mortality in patients hospitalized for COVID-19 in Rio de Janeiro, Brazil.

The baseline characteristics associated with mortality observed in this study were in line with the findings of other studies, such as a higher proportion of deaths in older patients, higher Charlson comorbidity index value, and history of stroke^3^ ^,^ ^9^ ^-^ ^10^. We also found a higher proportion of deaths in patients who lived in nursing homes (60%), in accordance with studies carried out in Brazil and other countries, which highlighted the high vulnerability of patients residing in nursing homes^11^.

All the clinical characteristics and laboratory parameters assessed have already been analyzed in several studies, although there is still no consensus on which are the most important^12^ ^-^ ^13^. We found that both leukocyte and neutrophil counts were significantly higher in the most severely affected patients who died, which may have been related to a secondary infection or infection-induced cytokine storm. In addition, these patients also had lower lymphocyte counts, although this was not significant. Lymphopenia is a common feature of many viral infections and may result from the direct infection of lymphocytes or cell apoptosis; therefore, monitoring the NLR in patients with COVID-19 has been suggested^12^.

We also found significantly higher levels of biomarkers of tissue and organ damage, such as LDH, AST, and urea. It has been postulated that this association could be explained by the virus causing direct damage to the organs by binding to angiotensin-converting enzyme 2 receptors; this leads to systemic hyperinflammation caused by a cytokine storm or hypoxia that results from respiratory failure^12^. In addition, as expected, due to the severity of the disease, higher APACHE II and SOFA scores, and lower values on the Glasgow scale were found in patients who died when compared to survivors.

Regarding the identified coinfections, in the literature, a higher frequency of complications and deaths have been described in critically ill patients with COVID-19 who had coinfection with influenza virus^14^. However, the same has not been reported in the studies published to date on coinfection with HIV^15^.

Despite having a small sample of patients, we found no statistically significant differences regarding the use of hydroxychloroquine, macrolides, ivermectin, low-molecular-weight heparin, or corticoids between the patients who survived and those who did not. Some of these observations were inconsistent with the findings of other studies, such as the protective effect of anticoagulants on the outcomes of patients with COVID-19.

Likewise, a greater use of antibiotics, although not statistically significant, was found in the group of patients who died. Ceftriaxone was used more frequently in both groups; however, carbapenems, linezolid, and polymyxin B were more frequently used in the group of patients who died. This may have been a reflection of the presence of secondary infections in this group of patients, especially in patients with ventilation-associated pneumonia.

This study showed that a SOFA score of ≥2 and an NLR of >5 on admission were independently associated with mortality in patients hospitalized for COVID-19, as has already been shown in studies from China, Europe, and the United States^12^ ^-^ ^13^. In future research, these parameters may serve to establish scores that will allow an initial assessment of patients with COVID-19 to provide timely care.

On the other hand, taking into account that the older adults made up almost the majority of the population in this study, we characterized a significant high-risk group that can benefit from stricter social distancing, especially when the restrictions due to COVID-19 are relaxed.

The present study had some limitations. First, this was a single-center study with a relatively small sample size, which was limited to the information recorded in the reports; therefore, some variables were not included in the analysis and their roles could have been underestimated. Second, patients who had incomplete information and who remained hospitalized until the end of the study period were excluded (data censored), which could have had an impact on the estimates. Third, the dynamic changes in laboratory parameters and their associations with mortality were not evaluated, and the clinical frailty of the patients was not evaluated. Finally, although the patient inclusion criteria were based on the clinical, radiological, and laboratory criteria of the Ministry of Health in Brazil, only 59% of the patients were confirmed by real-time RT-PCR, which could be linked to late hospitalization.

## Figures and Tables

**SUPPLEMENTARY FIGURE 1:**
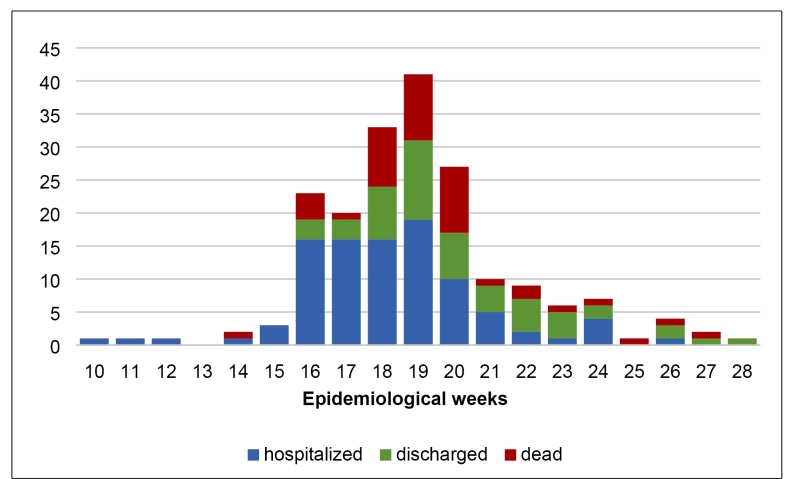
Hospitalizations and outcomes of the patients by epidemiological weeks.

**SUPPLEMENTARY FIGURE 2:**
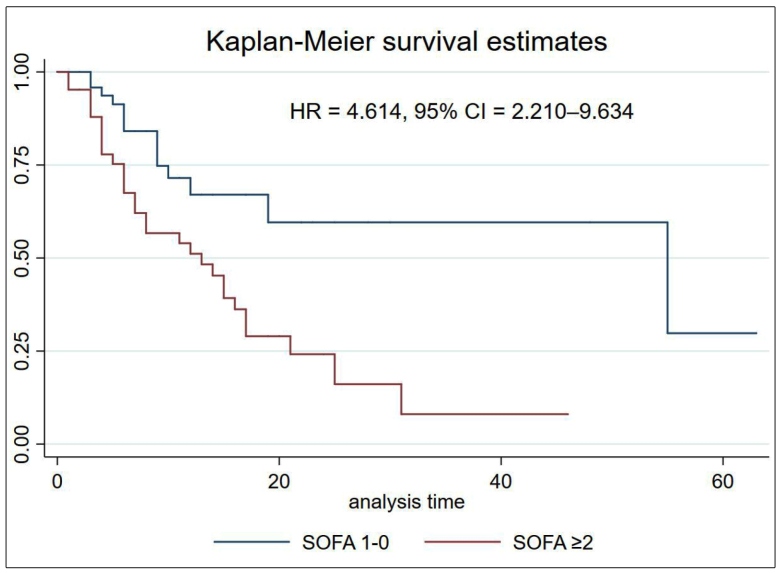
Kaplan-Meier curves of the survival analysis by the Sequential Organ Failure Assessment (SOFA) score. **HR:** hazard ratio; **CI:** confidence interval.

**SUPPLEMENTARY FIGURE 3:**
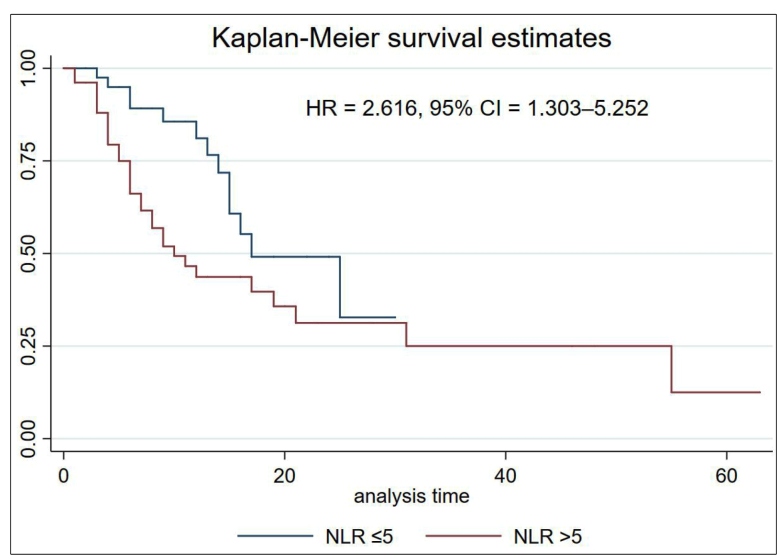
Kaplan-Meier curves of the survival analysis by the neutrophil/lymphocyte ratio (NLR). **HR:** hazard ratio; **CI:** confidence interval.

## SUPPLEMENTARY MATERIAL

**SUPPLEMENTARY TABLE 1:** Treatment and evolution of patients.

	Total		Survived		Died		p
	n=98		n=54		n=44		
**Treatment**							
# Antibiotics*	2	(2-3)	2	(2-3)	3	(2-4)	0.106
Hydroxychloroquine (n, %)	43	44%	19	35%	24	55%	0.065
Macrolides (n, %)	86	88%	48	89%	38	86%	0.704
Ivermectin (n, %)	21	21%	10	19%	11	25%	0.437
Corticosteroids (n, %)	12	12%	5	9%	7	16%	0.330
LMWH (n, %)	94	96%	51	94%	43	98%	0.625
Blood transfusion (n, %)	9	9%	2	4%	7	16%	0.074
Hemodialysis	37	38%	4	7%	33	75%	0.561
Mechanical ventilation	44	45%	3	6%	41	93%	**<0.001**
**Evolution**							
Length of stay*	9	(4-15)	10	(5, 17)	7	(4, 14)	0.220
Sepsis (n, %)	44	45%	1	2%	43	98%	**<0.001**
Septic shock (n, %)	43	44%	0	0%	43	98%	**<0.001**
ARDS	42	43%	2	4%	40	91%	**<0.001**
Acute renal failure (n, %)	50	51%	13	24%	37	84%	**<0.001**
Acute liver failure (n, %)	29	30%	6	11%	23	52%	**<0.001**
DIC	16	16%	0	0%	16	36%	**<0.001**
Encephalitis (n, %)	12	12%	0	0%	12	27%	**<0.001**

(*) median (IQR); **LMWH:** low-molecular-weight heparin; **ARDS:** acute respiratory distress syndrome; **DIC:** disseminated intravascular coagulation.
